# Cataract surgery without a phacoemulsification device during a humanitarian mission in benin: outcomes of manual small-incision cataract surgery and extracapsular cataract extraction

**DOI:** 10.1186/s12886-026-05148-2

**Published:** 2026-07-22

**Authors:** Ibrahim Ethem AY, Selim Genc, Chakiratou Olaïdé Adouké Abouki, Seray Yorukoglu Kayabas, Bilge Mumyakmaz Yaman

**Affiliations:** 1 Department of Ophthalmology, Faculty of Medicine, Afyonkarahisar Health Sciences University, Afyonkarahisar, Türkiye; 2 Department of Ophthalmology, Faculty of Medicine, İstanbul Okan University, İstanbul, Türkiye; 3 Department of Ophthalmology, Faculty of Medicine, Faculty of Health Sciences, University of Abomey-Calavi, Abomey-Calavi, Benin; 4 Department of Ophthalmology, Afyonkarahisar State Hospital, Afyonkarahisar, Türkiye

**Keywords:** Aphakia, Cataract, Extracapsular cataract extraction, Humanitarian mission, Manual small-incision cataract surgery, Low-resource settings, Phacoemulsification

## Abstract

**Background:**

To describe preoperative triage and compare early postoperative complications after manual small-incision cataract surgery (MSICS) and extracapsular cataract extraction (ECCE) performed during a humanitarian mission in Benin, where no phacoemulsification device was available.

**Methods:**

Medical records and operative reports from 1,280 individuals examined during a humanitarian cataract mission in Porto-Novo, Benin, between November 25 and December 6, 2024, were retrospectively reviewed. Patients were triaged according to cataract-related best-corrected visual acuity, systemic risk factors, and ocular findings. After exclusion of patients with capillary glucose > 300 mg/dL, blood pressure > 200/120 mmHg, or preoperative retinal detachment, 287 patients were considered suitable for surgery and 125 underwent surgery. Patients were classified by technique as MSICS (*n* = 79) or ECCE (*n* = 46). Postoperative complications documented on postoperative day 1 or during the mission were compared using two-sided Fisher exact tests.

**Results:**

Among the 125 operated patients, 79 underwent MSICS and 46 underwent ECCE. Aphakia occurred in 6 patients (4.8%), IOL dislocation in 3 (2.4%), marked endothelial edema in 11 (8.8%), postoperative IOP elevation in 12 (9.6%), and corneal suture dehiscence in 1 (0.8%). Marked endothelial edema was more frequent after ECCE than MSICS (8/46 [17.4%] vs. 3/79 [3.8%]; *p* = 0.018). No significant between-group differences were observed for aphakia (*p* = 0.192), IOL dislocation (*p* = 0.554), IOP elevation (*p* = 0.533), or corneal suture dehiscence (*p* = 0.368). No cases of IOL drop into the vitreous cavity, nuclear drop, intravitreal hemorrhage, toxic anterior segment syndrome, or endophthalmitis were observed in either group.

**Conclusion:**

In settings without a phacoemulsification device, MSICS and ECCE can be performed during humanitarian missions when experienced surgeons, careful preoperative triage, and appropriate postoperative assessment are available. MSICS may offer practical advantages where follow-up is limited because it generally avoids routine corneal suture removal. Because technique and surgeon were not independently separable in this nonrandomized study, the observed differences should not be interpreted as purely technique-related.

## Introduction

Cataract remains the leading cause of preventable blindness worldwide, and surgery is the definitive treatment for this condition [1]. Globally, approximately 1.1 billion people are estimated to be living with blindness or moderate-to-severe visual impairment, with cataract accounting for nearly half of these cases [2]. The development and widespread adoption of phacoemulsification have substantially improved surgical outcomes, making cataract treatment highly effective. Nevertheless, particularly in Africa, the diagnosis and management of cataract continue to represent major challenges in low-income countries. For example, among individuals aged > 50 years, the prevalence of bilateral blindness attributable to cataract has been reported to be 6.0% in countries of western sub-Saharan Africa and 5.7% in those of eastern sub-Saharan Africa [3]. To overcome barriers to accessing qualified healthcare services in these regions, numerous countries, including Türkiye, have organized humanitarian medical missions.

Notably, even at the tertiary hospital in the capital city of Benin, a country in western sub-Saharan Africa, where we participated in a Turkish humanitarian mission to perform cataract surgery, no phacoemulsification device was available. Therefore, in the present study, we aimed to report and analyze the demographic characteristics of patients, including the prevalence of bilateral blindness and associated comorbidities, as well as the outcomes of cataract surgeries performed using surgical techniques that predated phacoemulsification. By presenting these findings, we hope to provide practical guidance for volunteer ophthalmologists planning to perform cataract surgery in low-resource settings.

## Methods

### Study design and setting

This retrospective study reviewed the medical records and operative notes of patients who underwent cataract surgery between November 25 and December 6, 2024, in Porto-Novo, the capital of the Republic of Benin, as part of a humanitarian project supported by the Turkish Cooperation and Coordination Agency.

The cataract surgeries were organized in collaboration with the Turkish humanitarian team and were performed by two experienced ophthalmic surgeons (S.G. and İ.E.A.) in the ophthalmic operating room of a tertiary hospital in Porto-Novo. For the present analysis, patients were classified according to surgical technique: Group 1, manual small-incision cataract surgery (MSICS; *n* = 79), and Group 2, extracapsular cataract extraction (ECCE; *n* = 46). Each surgeon performed the technique in which they had the greatest experience; S.G. performed MSICS and İ.E.A. performed ECCE. Thus, surgeon and surgical technique were not independently separable. For study purposes, legal blindness was operationally defined as best-corrected visual acuity (BCVA) worse than 20/200 in the cataractous eye. Bilateral blindness was defined as BCVA worse than 20/200 in both eyes. Surgical indication was based on this visual acuity threshold. During triage, surgical priority was given to pediatric patients, individuals younger than 40 years of age, and patients with bilateral cataracts.

All patients underwent a comprehensive ophthalmologic examination, and ocular ultrasonography was performed when clinically indicated. Following the decision to proceed with cataract surgery, capillary blood glucose levels were measured by local healthcare personnel using a fingerstick test, and upper-arm systemic arterial blood pressure was measured. Measurements were repeated after a period of rest. Because access to cataract surgery in the region was limited and the mission represented a rare opportunity for treatment, pragmatic field thresholds were used: surgery was deferred in patients with capillary blood glucose levels exceeding 300 mg/dL or repeated blood pressure readings greater than 200/120 mmHg. These values were operational mission thresholds and were not intended to represent standard elective cataract-surgery safety limits. Patients younger than 18 years of age underwent general anesthesia, whereas all other patients underwent retrobulbar anesthesia using 2 mL per eye of Jetokain (2% lidocaine with epinephrine 0.0125 mg/mL). To facilitate communication and patient cooperation during local anesthesia, a local interpreter wearing sterile attire remained present in the operating room throughout surgery.

Because no phacoemulsification device was available at the hospital, cataract surgeries were performed using conventional extraction techniques that predated phacoemulsification. Although optical biometry was unavailable, IOL power calculations were performed locally using A-scan biometry. MSICS was performed through a scleral tunnel, whereas ECCE was performed through a superior corneal incision. Both techniques were performed by the surgeon with the greatest experience in that technique.

### Surgical technique 1: manual small-incision cataract surgery (MSICS)

Following adequate anesthesia, a side-port incision was created, and the anterior capsule was stained with trypan blue. After the dye was thoroughly irrigated from the anterior chamber using balanced salt solution, a sodium hyaluronate ophthalmic viscosurgical device (Hyotek 3% or Hyotek 1.4%) was injected. A continuous curvilinear capsulorhexis was then performed using a cystotome. When a continuous circular capsulorhexis could not be achieved, a can-opener capsulotomy technique was employed.

The superior conjunctiva was opened and dissected with scissors over an arc of approximately 80°–90°. A curvilinear scleral incision measuring approximately 6–7 mm in length and extending to half the scleral thickness was created 1.5 mm posterior to the limbus using a 45° blade. From the apex of this incision, a scleral tunnel was dissected toward the clear cornea with a crescent blade and subsequently extended in both directions to complete tunnel formation. The tunnel was then entered into the anterior chamber using a 3.2-mm blade, and the internal incision was enlarged bilaterally to approximately 10 mm.

Following hydrodissection, additional ophthalmic viscosurgical device was injected into the anterior chamber, and the crystalline lens was extracted using a lens loop and curette. A second side-port incision was then created, and residual cortical material was removed using a Simcoe irrigation–aspiration cannula. A polymethyl methacrylate (PMMA) IOL was implanted into the ciliary sulcus through the main incision. The ophthalmic viscosurgical device was subsequently removed from the anterior chamber using a Simcoe cannula. Intracameral cefuroxime (Aprokam, 1 mg/0.1 mL) was administered at the conclusion of surgery.

The side-port incisions were sealed by stromal hydration. After confirming the watertight integrity of the main incision, an 8 − 0 Vicryl suture was placed in the sclera when deemed necessary. Following conjunctival closure with 8 − 0 Vicryl sutures, subconjunctival gentamicin (Genta, 40 mg/mL; 0.5 mL, corresponding to 20 mg) and dexamethasone (Dekort, 4 mg/mL; 0.5 mL, corresponding to 2 mg) were administered.

### Surgical technique 2: extracapsular cataract extraction (ECCE)

After adequate anesthesia was achieved, a side-port incision was created, and the anterior capsule was stained with trypan blue. Following removal of the capsular dye using balanced salt solution, a sodium hyaluronate ophthalmic viscosurgical device (Hyotek 3% or Hyotek 1.4%) was injected into the anterior chamber. A superior main incision was then created using a 2.8-mm blade. Continuous curvilinear capsulorhexis was performed with the aid of a cystotome and Utrata forceps; when a circular capsulorhexis could not be achieved, a capsulotomy was performed using the can-opener technique. The superior incision was subsequently enlarged in both directions with scissors to approximately 180°. After hydrodissection, additional ophthalmic viscosurgical device was injected into the anterior chamber, and the lens was delivered into the anterior chamber using a lens loop and curette. Residual cortical material was removed with a Simcoe cannula. A PMMA IOL was implanted in the sulcus through the main incision. The viscoelastic material was then aspirated from the anterior chamber using a Simcoe cannula. Depending on the density of the extracted cataract and the width of the incision, the main wound was secured with three, four, or five interrupted 10 − 0 polypropylene sutures. Intracameral cefuroxime (Aprokam, 1 mg/0.1 mL) was administered, and wound integrity was confirmed by assessing watertight closure. Finally, subconjunctival gentamicin (Genta, 40 mg/mL; 0.5 mL, corresponding to 20 mg) and dexamethasone (Dekort, 4 mg/mL; 0.5 mL, corresponding to 2 mg) were injected.

For both surgical techniques, anterior vitrectomy was unavailable in cases of intraoperative posterior capsule rupture. Therefore, sponge vitrectomy was routinely performed, and the procedure was completed by injecting sterile air into the anterior chamber. MSICS was performed using a Möller–Wedel microscope (Haag-Streit, Germany), whereas ECCE was performed using an Asslar microscope (Asslar/Wetzlar, Germany). Both surgeons were assisted by two operating-room nurses experienced in ophthalmic surgery. The nurse with greater experience in African field settings assisted during MSICS. Surgical instruments were sterilized under field conditions in Africa using flash autoclaving because no alternative sterilization method was available.

### Postoperative management and follow-up

Following surgery, all patients routinely received Moxai 0.5% (moxifloxacin 5 mg/mL) eye drops hourly for the first 2 days and then four times daily for 1 week. Pred Forte 1% (prednisolone acetate) was administered hourly during the first postoperative week and then tapered weekly. A biomicroscopic examination was performed on postoperative day 1 by the operating surgeons. Patients who developed complications or had clinically significant findings were recalled for follow-up evaluations as needed. Local hospital personnel performed scheduled follow-up visits at postoperative week 1 and postoperative months 1 and 3; these later observations were not included in the present dataset because the study analysis was based on postoperative day-1 documentation and mission records. Patients with an uncomplicated postoperative course received written information and were instructed, through a local interpreter, to return to the hospital should any problems arise. Patients who underwent corneal suturing were advised to return at postoperative month 3 for suture removal.

### Statistical analysis

All statistical analyses were performed using SPSS software (SPSS Inc., version 23.0, Chicago, IL, USA). Categorical variables were summarized as frequencies and percentages. For each complication parameter, the MSICS and ECCE groups were compared using a two-sided Fisher exact test. A two-sided p-value of < 0.05 was considered indicative of statistical significance.

## Results

**Fig. 1:**
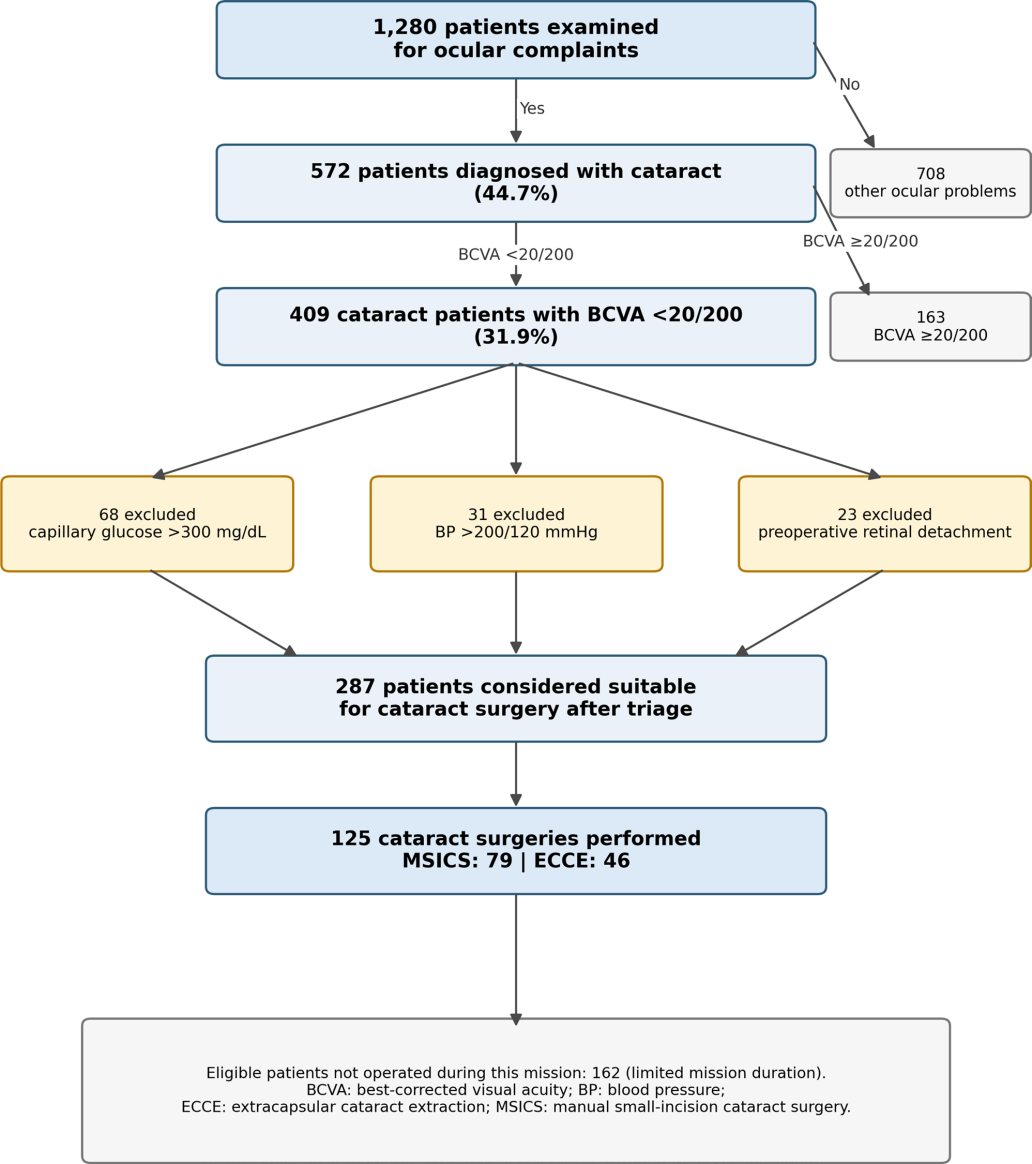
Patient triage flowchart during the humanitarian cataract mission. BCVA, best-corrected visual acuity; BP, blood pressure; ECCE, extracapsular cataract extraction; MSICS, manual small-incision cataract surgery

Among patients presenting to the hospital with visual complaints, a total of 1,280 individuals were examined, and cataract was diagnosed in 572 patients (44.7%). Of these patients, 163 (12.7%) had BCVA of ≥ 20/200 in the cataractous eye and did not meet the study-defined surgical threshold. Consequently, 409 patients (32.0%) were evaluated for cataract surgery. Among those considered potential surgical candidates, surgery was cancelled in 68 patients (5.3%) because repeated preoperative fingerstick blood glucose levels remained > 300 mg/dL after a period of rest. An additional 31 patients (2.4%) were excluded from surgery because repeated preoperative systemic blood pressure measurements exceeded 200/120 mmHg. Furthermore, 23 patients (1.8%) were not included in the surgical plan because preoperative ocular ultrasonography revealed retinal detachment and cataract surgery was not expected to provide visual benefit (Table 1; Fig. 1).

**Table 1:** Triage assessment of patients presenting for the cataract mission

Parameter	*n*	%
Number of patients examined preoperatively	1,280	100
Patients with ocular problems due to causes other than cataract (e.g., refractive error)	708	55.3
Number of patients diagnosed with cataract	572	44.7
Patients whose cataract did not reduce best-corrected visual acuity below the study-defined threshold*	163	12.7
Patients whose cataract reduced best-corrected visual acuity below the study-defined threshold**	409	32.0
Patients excluded from surgical planning because of elevated capillary blood glucose***	68	5.3
Patients excluded from surgical planning because of elevated blood pressure****	31	2.4
Patients excluded from surgical planning because of preoperative retinal detachment	23	1.8
Patients deemed suitable for cataract surgery	287	22.4
Total number of cataract surgeries performed*****	125	9.8

* Patients with cataract whose best-corrected visual acuity in the cataractous eye was ≥ 20/200 and who therefore did not meet the study-defined surgical threshold

** Patients with cataract whose best-corrected visual acuity in the cataractous eye was < 20/200 and who met the study-defined surgical threshold

*** Patients with repeated capillary blood glucose levels > 300 mg/dL after a period of rest

**** Patients with repeated upper-arm blood pressure readings > 200/120 mmHg after a period of rest

***** Patients who underwent cataract surgery during the mission following triage

Of the 125 patients who underwent surgery, 6 (4.8%) received general anesthesia because of their young age and a diagnosis of congenital cataract, whereas 119 (95.2%) underwent retrobulbar anesthesia because they were considered suitable candidates for local anesthesia. No clinically significant anesthesia-related complications were observed among patients who received general anesthesia. Among those who underwent retrobulbar anesthesia, retrobulbar hemorrhage accompanied by proptosis developed in 1 patient (0.8%). This complication was managed with cold compresses and firm eye closure; surgery was postponed for 3 days and was subsequently completed successfully. No serious complications associated with retrobulbar hemorrhage, such as scleral perforation, were observed.

A total of 68 patients had cataracts associated with severe visual impairment in both eyes, representing 54.4% of the study population. Regarding surgical approach, 79 patients (63.2%) underwent MSICS and 46 patients (36.8%) underwent ECCE.

Among patients who underwent MSICS, 2 (2.5%) were left aphakic, IOL dislocation occurred in 1 (1.3%), marked endothelial edema occurred in 3 (3.8%), and postoperative IOP elevation was observed in 9 (11.4%); no corneal suture dehiscence occurred. Among patients who underwent ECCE, 4 (8.7%) were left aphakic, IOL dislocation occurred in 2 (4.3%), marked endothelial edema occurred in 8 (17.4%), IOP elevation was detected in 3 (6.5%), and postoperative corneal suture dehiscence occurred in 1 (2.2%). Two-sided Fisher exact testing showed a significant between-group difference only for marked endothelial edema (*p* = 0.018); p-values for aphakia, IOL dislocation, IOP elevation, and corneal suture dehiscence were 0.192, 0.554, 0.533, and 0.368, respectively (Table 2).

**Table 2:** Distribution of complications according to cataract surgery technique

Complication	MSICS (*n* = 79)	ECCE (*n* = 46)	Total (*n* = 125)	*p*-value
Aphakia	2 (2.5%)	4 (8.7%)	6 (4.8%)	0.192
IOL dislocation	1 (1.3%)	2 (4.3%)	3 (2.4%)	0.554
Marked endothelial edema	3 (3.8%)	8 (17.4%)	11 (8.8%)	0.018
Postoperative IOP elevation	9 (11.4%)	3 (6.5%)	12 (9.6%)	0.533
Corneal suture dehiscence	0 (0.0%)	1 (2.2%)	1 (0.8%)	0.368

* Marked endothelial edema was defined as endothelial edema extending both horizontally and vertically across the cornea in the postoperative period. Of the 11 patients with marked endothelial edema, 3 underwent MSICS and 8 underwent ECCE

** IOP elevation was defined as a postoperative IOP > 25 mmHg. Among the 3 patients with elevated IOP who underwent ECCE, 1 was aphakic; among the 9 patients who underwent MSICS, 2 were aphakic

*** Corneal suture dehiscence was defined as postoperative separation of a previously placed corneal wound suture and occurred in 1 patient in the ECCE group

Note: P-values were calculated using two-sided Fisher exact tests

Aphakic patients and those with IOL dislocation were followed up regularly. In patients with marked endothelial edema, topical 3% sodium chloride drops were prepared and administered four times daily. In patients with elevated IOP, Tomec (dorzolamide 2%/timolol 0.5%) was administered twice daily and proved effective. The patient with corneal suture dehiscence underwent same-day resuturing and was subsequently followed without further complications. In both surgical techniques, no serious complications were observed, including IOL drop into the vitreous cavity, nuclear drop into the vitreous, intravitreal hemorrhage, toxic anterior segment syndrome, or endophthalmitis.

## Discussion

In Africa, cataract is the most common cause of preventable blindness, affecting not only the elderly but also all age groups, particularly children. As countries become more developed, posterior segment diseases tend to replace cataract and emerge as more prominent causes of blindness. However, in Africa, even in relatively developed settings, cataract remains an incompletely resolved public health problem [4–8]. Reported cataract prevalence across the continent includes 54.1% in Uganda, 47.8% in Mali, 44.5% in Eritrea, 31.6% in The Gambia, and 19.4% in Tanzania [9–11]. In Benin, where we participated in a humanitarian mission, no published study reporting cataract prevalence was identified in the literature. Nevertheless, cataract surgery is widely recognized as one of the most cost-effective surgical interventions due to its substantial benefits for individual health and social participation [12]. Unfortunately, in Benin and many similar low-income countries, this burden remains unresolved, and continued support from volunteer medical teams from other countries is still required.

In the region where cataract surgeries were performed as part of a humanitarian mission, 1,280 individuals presented to the local hospital with ocular complaints over a short period. Patients with refractive errors, ocular itching, and similar conditions were identified and referred to local ophthalmologists for management and follow-up. Following routine ophthalmologic examination, patients with cataract were prioritized for surgery based on predefined criteria, including BCVA worse than 20/200 in the cataractous eye, pediatric age, bilateral cataract, and monocular status. During preoperative evaluation, surgery was cancelled in patients with repeated capillary blood glucose levels > 300 mg/dL or repeated systemic arterial blood pressure measurements > 200/120 mmHg after rest. These patients were referred to the appropriate hospital departments for further management. Indeed, several studies have demonstrated that uncontrolled diabetes impairs wound healing, promotes bacterial proliferation, and significantly increases the risk of endophthalmitis [13–15]. In humanitarian missions conducted in Africa, comprehensive preoperative support from local tertiary hospitals may not always be available. When available, systemic screening for uncontrolled diabetes and hypertension is beneficial in improving surgical safety.

We found no studies in the literature comparing MSICS and ECCE in humanitarian cataract surgery missions conducted in low-income countries where phacoemulsification technology is unavailable. In our analysis, marked endothelial edema was observed less frequently after MSICS than after ECCE (3.8% vs. 17.4%; *p* = 0.018), whereas no significant between-group differences were detected for the other complications. This comparison must nevertheless be interpreted cautiously. Each technique was performed by a different surgeon, and the operating microscopes and assistant experience differed between the groups. Therefore, surgeon-, equipment-, and technique-related effects cannot be separated, and the findings should be considered an exploratory technique-stratified analysis rather than a randomized head-to-head comparison.

Because a local tertiary hospital capable of providing long-term postoperative follow-up was available in the region where this study was conducted, the use of sutures in the ECCE group may not be expected to pose a major problem. However, in similar surgeries performed in rural regions of Africa, MSICS may be more appropriate because it generally does not require suture removal. Indeed, the literature has reported that not only sutures retained for prolonged periods but even those removed at the appropriate time can lead to endophthalmitis [16, 17]. In field-based surgery of this type, surgeons should use the technique with which they have the greatest experience, while recognizing that the present data cannot establish the independent superiority of either technique. In settings where vitrectomy is not available, complications such as dropped nucleus, dropped IOL, retinal detachment, and endophthalmitis are considerably more difficult to manage than under standard conditions.

It was reassuring that no such serious complications occurred during the mission. The surgeries were performed simultaneously by two surgeons at two separate operating tables within the same operating room. Moreover, the operating room was not equipped with a high-efficiency particulate air (HEPA) filter, and cooling was provided only by a standard office-type air-conditioning unit. The literature indicates that the use of HEPA filtration in operating rooms reduces the risk of endophthalmitis [18]. Sterilization procedures were performed using flash autoclaving because this was the only sterilization method available in the field setting. Reference 19 lists ethylene oxide, autoclave, flash autoclave, and plasma sterilizer as preferred sterilization options and recommends autoclave sterilization between cases with appropriate documentation. We therefore clarify that flash autoclaving in our mission was a resource-driven necessity and was not intended to imply that it is universally preferred over validated full-cycle sterilization.

Considering that patients may be more susceptible to infection because of living conditions, poor adherence to eye-drop regimens, language barriers, and low educational levels, the absence of endophthalmitis in our study was encouraging. Intracameral cefuroxime (1 mg/0.1 mL) was administered as prophylaxis. Both intracameral moxifloxacin and cefuroxime have been evaluated in cataract surgery literature [20, 21], but the present study was not designed to determine the preventive effect of cefuroxime. The use of postoperative topical antibiotic and steroid therapy, together with careful triage and day-1 examination, may have contributed to the favorable early safety profile, although causal inferences cannot be made. The glucose and blood-pressure thresholds used in this mission were deliberately pragmatic because access to future cataract surgery was limited; they should not be generalized to routine elective cataract surgery. Current guidance recommends lower preoperative values, including fasting glucose ≤ 140 mg/dL, random or postprandial glucose ≤ 200 mg/dL, and blood pressure ≤ 160/95 mmHg [19].

One limitation of our study was that, due to the limited duration of the field mission, surgery could not be performed in all patients who presented to the hospital and were deemed to require cataract surgery. Information on these patients was recorded by the local hospital, and they were informed that priority would be given to them in future missions. This situation clearly reflects the substantial unmet need for cataract surgery in the region and, at the same time, highlights limited access to health care in low-income countries and the resulting patient burden.

The retrospective design of our study constitutes an important limitation. The sex of the patients could not be reliably determined retrospectively from the names recorded in the patient files; therefore, the sex distribution of the operated individuals could not be established. In addition, age data were frequently missing from the records. During the field mission, we also observed that many patients did not know their exact age; therefore, age distribution was excluded from the demographic analysis. The refractive outcomes of surgery, the accuracy of biometry measurements, and late postoperative complications could not be assessed because the follow-up examinations performed by local personnel at postoperative week 1 and months 1 and 3 were not available in the study dataset. Furthermore, because each technique was performed by a different surgeon and with different microscope and assistant conditions, the independent effects of surgical technique and surgeon could not be separated. The analysis was also limited to major complications documented on postoperative day 1 or during the mission. Despite these limitations, given the scarcity of literature in this field and the numerous challenges inherent in participating in humanitarian missions of this nature, we believe that the data obtained from the available records remain meaningful and may serve as a guide for ophthalmologists participating in similar missions. Prospective multicenter studies with standardized surgery and follow-up are warranted.

## Conclusions

Cataract surgeries performed for humanitarian purposes in low-income countries may be conducted under conditions that differ substantially from the surgical standards available in high-income settings. Limited ophthalmic experience among operating-room staff, the need to perform procedures in patients who speak different languages, resource-driven sterilization constraints, incomplete access to postoperative follow-up, and limited imaging capacity of operating microscopes are among the factors that may increase complication rates in such missions. Therefore, cataract surgery under these conditions should be performed by experienced surgeons, preoperative assessment and patient triage should be meticulous, and limited field time should be used efficiently. When no phacoemulsification device is available, MSICS and ECCE remain feasible approaches, but their outcomes should be interpreted in the context of surgeon and resource-related confounding.

## Data Availability

The datasets used and/or analysed during the current study are available from the corresponding author on reasonable request.
